# Pyramids of QTLs enhance host–plant resistance and Bt-mediated resistance to leaf-chewing insects in soybean

**DOI:** 10.1007/s00122-015-2658-y

**Published:** 2016-01-02

**Authors:** María A. Ortega, John N. All, H. Roger Boerma, Wayne A. Parrott

**Affiliations:** Institute of Plant Breeding, Genetics and Genomics, University of Georgia, 111 Riverbend Rd., Athens, GA 30602 USA; Department of Entomology, University of Georgia, 413 Biological Sciences Building, Athens, GA 30602 USA

## Abstract

****Key message**:**

**QTL-M and QTL-E enhance soybean resistance to insects. Pyramiding these QTLs with*cry1Ac* increases protection against Bt-tolerant pests, presenting an opportunity to effectively deploy Bt with host–plant resistance genes**.

**Abstract:**

Plant resistance to leaf-chewing insects minimizes the need for insecticide applications, reducing crop production costs and pesticide concerns. In soybean [*Glycine max* (L.) Merr.], resistance to a broad range of leaf-chewing insects is found in PI 229358 and PI 227687. PI 229358’s resistance is conferred by three quantitative trait loci (QTLs): M, G, and H. PI 227687’s resistance is conferred by QTL-E. The letters indicate the soybean Linkage groups (LGs) on which the QTLs are located. This study aimed to determine if pyramiding PI 229358 and PI 227687 QTLs would enhance soybean resistance to leaf-chewing insects, and if pyramiding these QTLs with Bt (*cry1A*c) enhances resistance against Bt-tolerant pests. The near-isogenic lines (NILs): Benning^ME^, Benning^MGHE^, and Benning^ME+*cry1Ac*^ were developed. Benning^ME^ and Benning^MGHE^ were evaluated in detached-leaf and greenhouse assays with soybean looper [SBL, *Chrysodeixis includens* (Walker)], corn earworm [CEW, *Helicoverpa zea* (Boddie)], fall armyworm [FAW, *Spodoptera frugiperda* (J.E. Smith)], and velvetbean caterpillar [VBC, *Anticarsia gemmatalis* (Hübner)]; and in field-cage assays with SBL. Benning^ME+*cry1Ac*^ was tested in detached-leaf assays against SBL, VBC, and Southern armyworm [SAW, *Spodoptera eridania* (Cramer)]. In the detached-leaf assay, Benning^ME^ showed the strongest antibiosis against CEW, FAW, and VBC. In field-cage conditions, Benning^ME^ and Benning^MGHE^ suffered 61 % less defoliation than Benning. Benning^ME+*cry1Ac*^ was more resistant than Benning^ME^ and Benning^*cry1Ac*^ against SBL and SAW. Agriculturally relevant levels of resistance in soybean can be achieved with just two loci, QTL-M and QTL-E. ME+*cry1Ac* could present an opportunity to protect the durability of Bt genes in elite soybean cultivars. These results should assist the development of effective pest management strategies, and sustainable deployment of Bt genes in soybean.

## Introduction

The production of soybean [*Glycine max* (L.) *Merr*], one of the world’s primary sources of vegetable oil and protein (Wilcox 2004), is often limited by pests. Worldwide, 11 % of the crop is lost to animal pests, including insects (Oerke 2005). In the USA, the insect pests causing the most impact are: corn earworm [*Helicoverpa zea* (Boddie)], soybean looper [*Chrysodeixis includens* (Walker)], velvetbean caterpillar [*Anticarsia gemmatalis* (Hübner)], bean leaf beetle, [*Cerotoma trifurcata* (Forster)], green stink bug [*Chinavia hilaris* (Say)], and southern stink bug [*Nezara viridula* (L)] (Boethel 2004). The corn earworm (CEW), soybean looper (SBL), velvetbean caterpillar (VBC), and bean leaf beetle are chewing insects capable of defoliating plants entirely. Although soybean plants can withstand moderate levels of leaf damage, high levels of defoliation greatly reduce seed yield and quality (Haile et al. 1998). The efficient use of insecticide applications depends on economic thresholds (ETs), which are based on percent of defoliation and are used to monitor insect populations to prevent them from reaching levels that may cause economic losses. The suggested ETs for leaf-chewing insects in soybean are 35 % defoliation during the vegetative stages and 20 % defoliation during the reproductive stages (Heatherly 2014).

A third of the world’s soybean crop was produced in the USA in 2013 (FAOSTAT 2015). The southern states of Alabama, Arkansas, Louisiana, Mississippi, North Carolina, Tennessee, and Virginia harvested just 13.6 % of the US supply; yet farmers in these states spent $262 million on insect control to produce a $5 billion crop. Despite the control efforts, yield losses to insects amounted to $234 million. Thus, the combined costs of insect control and yield loss were equivalent to $500 million. CEW, SBL, and stink bugs were the most important species, both in terms of control costs and yield losses (Musser et al. 2014). The need to lower cost of production along with increased concern over insecticide residues in the food chain and environment is incentives to develop insect-resistant cultivars to use in integrated pest management (IPM) strategies. However, these efforts have been hampered by a lack of understanding of the genetic basis of resistance to most insects, in addition to the difficulty of developing insect-resistant cultivars that yield equivalently to the existing cultivars (Lambert and Tyler 1999).

The Japanese soybean landraces ‘Kosamame’ (PI 171451), ‘Miyako White’ (PI 227687), and ‘Sodendaizu’ (PI 229358) are the most widely used sources of resistance to defoliating insects (USDA-ARS 2015). They were initially discovered to be resistant to Mexican bean beetle [*Epilachna varivestis* (Mulsant)] by Van Duyn et al. (1971, 1972). They also have been reported to be resistant to multiple coleopteran, lepidopteran, and hemipteran insects that are major economic pests of soybean worldwide (Clark et al. 1972; Gary et al. 1985; Hatchett et al. 1976; Hoffmann-Campo et al. 2006; Jones and Sullivan 1979; Komatsu et al. 2004; Lambert and Kilen 1984b; Layton et al. 1987; Li et al. 2008; Luedders and Dickerson 1977; Piubelli et al. 2003; Silva et al. 2013; Talekar and Lee 1988; Talekar and Lin 1994).

Resistance to defoliating insects in PI 171451, PI 227687, and PI 229358 is conferred via both antibiosis and antixenosis (Rector et al. 2000a, b). Antibiosis is a type of resistance in which the plant has a detrimental effect on insect growth, development, and/or reproduction (Painter 1951). Antixenosis or non-preference is a type of resistance in which the plant affects insect behavior, by discouraging oviposition, colonization, or feeding (Kogan and Ortman 1978; Painter 1951). Initial attempts to transfers insect resistance from these plant introductions (PIs) to elite soybean lines were hindered by poor agronomic qualities of the PIs, and by quantitative inheritance of resistance (Boethel 1999). The advent of marker-assisted selection (MAS) has made possible to reduce many of the issues caused by linkage drag (Warrington et al. 2008).

To understand the genetic basis of resistance in these PIs, Rector et al. (1998, 2000a, b) identified a major QTL on Linkage Group (LG) M (now chromosome 7) of PI 171451 and PI 229358. This locus named “QTL-M” accounts for 37 % of antixenosis variance, and up to 28 % of antibiosis variance. In addition, there are two minor QTLs involved in resistance. QTL-H on chromosome (formerly LG H) conditions antixenosis in PI 229358 and PI 171451, and QTL-G on chromosome 18 (formerly LG G) conditions antibiosis in PI 229358. Zhu et al. (2006) demonstrated that QTL-H, and QTL-G only have a detectable effect if QTL-M is present in the genome. These minor QTLs have usually been missed by conventional breeding programs (Narvel et al. 2001).

Hulburt (2002) identified a major insect-resistance QTL in a mapping population from a PI 227687 × ‘Cobb’ cross. This QTL (QTL-E) on LG E (now chromosome 12) of PI 227687 conveys both antibiosis and antixenosis. QTL-E co-maps with the *Pb* locus that controls sharp (*Pb_*) vs. blunt (*pbpb*) leaf pubescence in soybean (Ting 1946). Although there are earlier reports on the effect of pubescence traits on soybean resistance to insect (Hollowell and Johnson 1934; Johnson and Hollowell 1935; Kanno 1996), is the first report that a sharp-trichome locus co-localizes with an insect-resistance QTL. Hulburt et al. (2004) confirmed that sharp-trichome NILs from ‘Clark’ and ‘Harosoy’ are more resistant to lepidopterans, compared to the blunt-trichome cultivars. Nevertheless, given that Lambert and Kilen (1984b) showed that PI 227687’s resistance is graft-transmissible, it remains possible that resistance is really due to an as of yet unidentified gene linked to *Pb*.

Pyramiding is used to combine multiple desirable genes for the same trait into a single genetic background (Ye and Smith 2008). This strategy is advantageous for development of insect-resistant cultivars; it permits genes with different modes of action to be combined to obtain more durable resistance. Accordingly, Walker et al. (2002) demonstrated that QTL-M enhances the effectiveness of Bt in soybean plants expressing the *cry1Ac* transgene, while Santos et al. (1997) found that the use of cowpea trypsin inhibitor countered the effects of Cry1Ac in arabidopsis. In addition, Zhu et al. (2008) analyzed sixteen NILs carrying all possible combinations of the insect-resistance QTLs from PI 229358 and the *cry1Ac* transgene in a ‘Benning’ background (Boerma et al. 1997). CEW and SBL bioassays confirmed that Cry1Ac is more effective in the presence of insect-resistance QTLs from PI 229358.

The main goal of this research is to enhance soybean resistance to leaf-chewing insects by identifying the best combination of host–plant resistance QTLs. The objectives of this study were to: (1) develop NILs containing novel combinations of the insect-resistance QTLs from PI 229358 and PI 227687; (2) characterize the NILs for their resistance to defoliating insects, and (3) evaluate the effect of the combination of QTL-M, QTL-E, and Bt for controlling Bt-tolerant pests.

## Materials and methods

### Characterization of Benning^ME^ and Benning^MGHE^

#### Development of near-isogenic lines

**Fig. 1:**
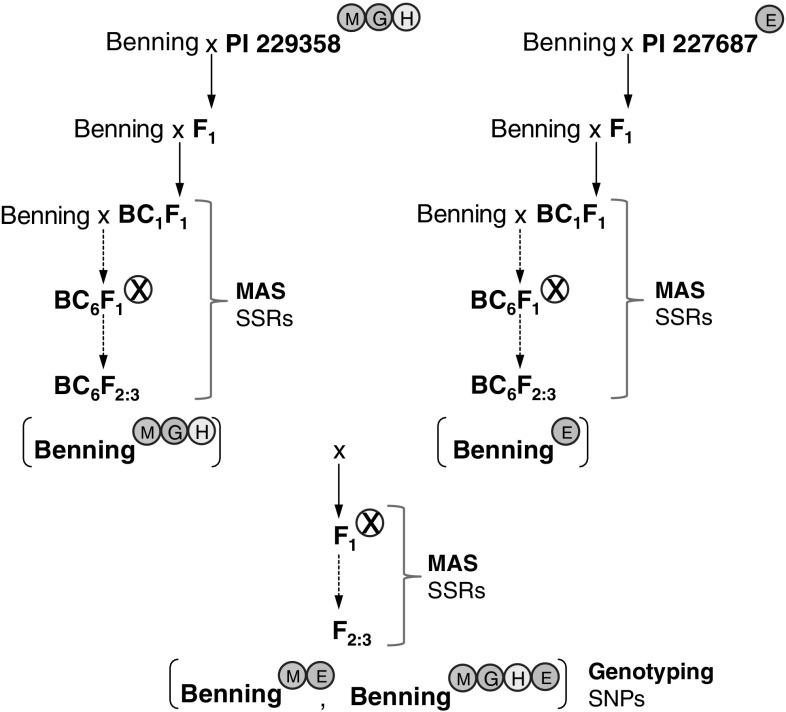
Breeding scheme for pyramiding insect-resistance QTLs in Benning. Benning^MGH^ (Zhu et al. 2007) and Benning^E^, developed from a cross between Benning and PI 227687, were crossed; and the QTL combinations Benning^ME^ and Benning^MGHE^ were selected in the progeny. SSRs were used for marker-assisted selection (MAS) of QTL pyramids in each generation, and SNPs (Ortega 2016, personal communications) were used to genotype the plants used in the bioassays

The BC_6_F_2_-derived NILs, Benning^ME^ and Benning^MGHE^ [i.e., Benning with QTLs M and E in the first case and M, G, H, and E in the second case, backcrossed into it] were developed using a marker-assisted backcross approach (Fig. 1). Benning, a Maturity Group VII elite cultivar adapted to Georgia, was used as the recurrent parent. The NIL development took approximately 10 years, and started before SNPs were commonplace. Simple sequence repeat (SSR) markers linked to each QTL were used during backcross and selfing generations to select lines carrying a specific QTL combination. The flanking markers were: Sat_258 (5′-GCGCAATAGATAATCGAAAAACATACAAGA-3′ and 5′-GCGGGGAAAGTGAAAACAAGATCAAATA-3′) and Satt702 (5′-GCGGGGTTCTGTGGCTTCAAC-3′ and 5′-GCGCATTGGAATAACGTCAAA-3′) for QTL-M (Zhu et al. 2009); Sct_199 (5′-GCGACAATGGCTATTAGTAACAATCA-3′ and 5′-GCGATTTTCTATTTTCCTCACAGTG-3′) and Satt191 (5′-CGCGATCATGTCTCTG-3′ and 5′-GGGAGTTGGTGTTTTCTTGTG-3′) for QTL-G (Zhu et al. 2008); Sat_334 (5′-GCGTAACGTAGCAAATTGACTATAAGA-3′ and 5′-GCGTGTGCAAAGACAATTTCAATGA-3′) and Sat_122 (5′-GTGACAAATGGATGGACAATAG-3′ and 5′-AAGAAAAATAAAATAATGTAGAGTGGTGAT-3′) for QTL-H (Zhu et al. 2008); and Sat_112 (5′-TGTACAGTATACCGACATAATA-3′ and 5′-CTACAAATAACATGAAATATAAGAAATA-3′) and Satt411 (5′-TGGCCATGTCAAACCATAACAACA-3′ and 5′-GCGTTGAAGCCGCCTACAAATATAAT-3′) for QTL-E (Hulburt 2002). Primer sequences for the SSR markers were obtained from SoyBase (http://www.soybase.org) (Grant et al. 2010). Genomic DNA isolation, PCR, and electrophoresis protocols for SSRs were performed as described by Zhu et al. (2008). Single nucleotide polymorphism (SNP) markers (Ortega 2016, personal communications) were used to genotype the plants used in the bioassays.

#### Defoliation

**Fig. 2:**
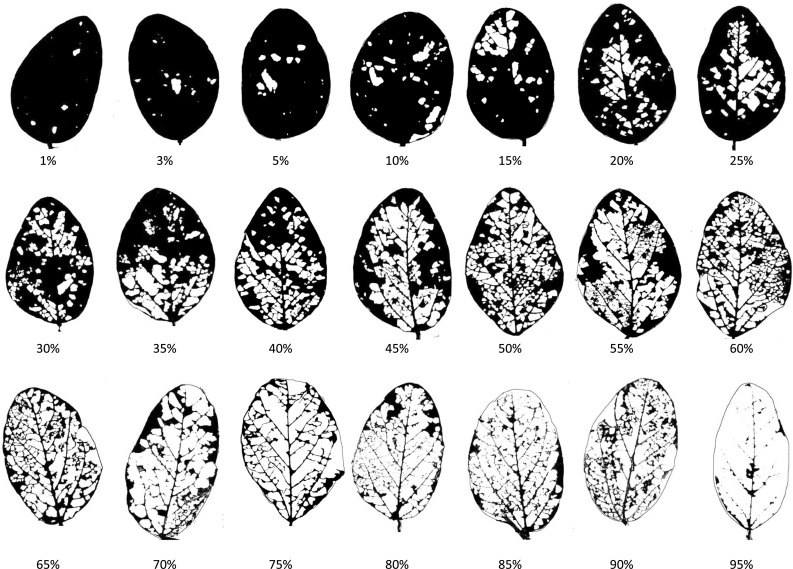
Soybean defoliation chart. Percentage of leaf area consumed by herbivores was calculated using Image J

To estimate defoliation percentage, a soybean leaf defoliation chart (Fig. 2) was built from a collection of chewed leaves for which the percentage of consumed leaf area was calculated in Image J (Rasband 1997). A chart including 5 % increments was the most useful to estimate the percent defoliation in NILs carrying the minor insect-resistance QTLs (QTL-G and QTL-H) in combination with the major QTLs (QTL-M and QTL-E).

#### Bioassays

SBL, CEW, fall armyworm [*Spodoptera frugiperda* (J.E. Smith)], and VBC caterpillars were used to evaluate the insect-resistant NILs performance in antibiosis, antixenosis, and field-cage assays. Eggs were obtained from Benzon Research Inc. (Carlisle, PA). Eggs were incubated for 72 h at 25 °C in a 600-ml (20 oz) clear polystyrene cup (Letica Corporation, Rochester Hills, MI, USA) sealed with a dome lid (Letica Corporation); the cup contained 7 ml of plaster of Paris saturated with water to maintain 75 % relative humidity. Neonate caterpillars were used to infest the bioassays.

##### Detached-leaf experiments

**Fig. 3:**
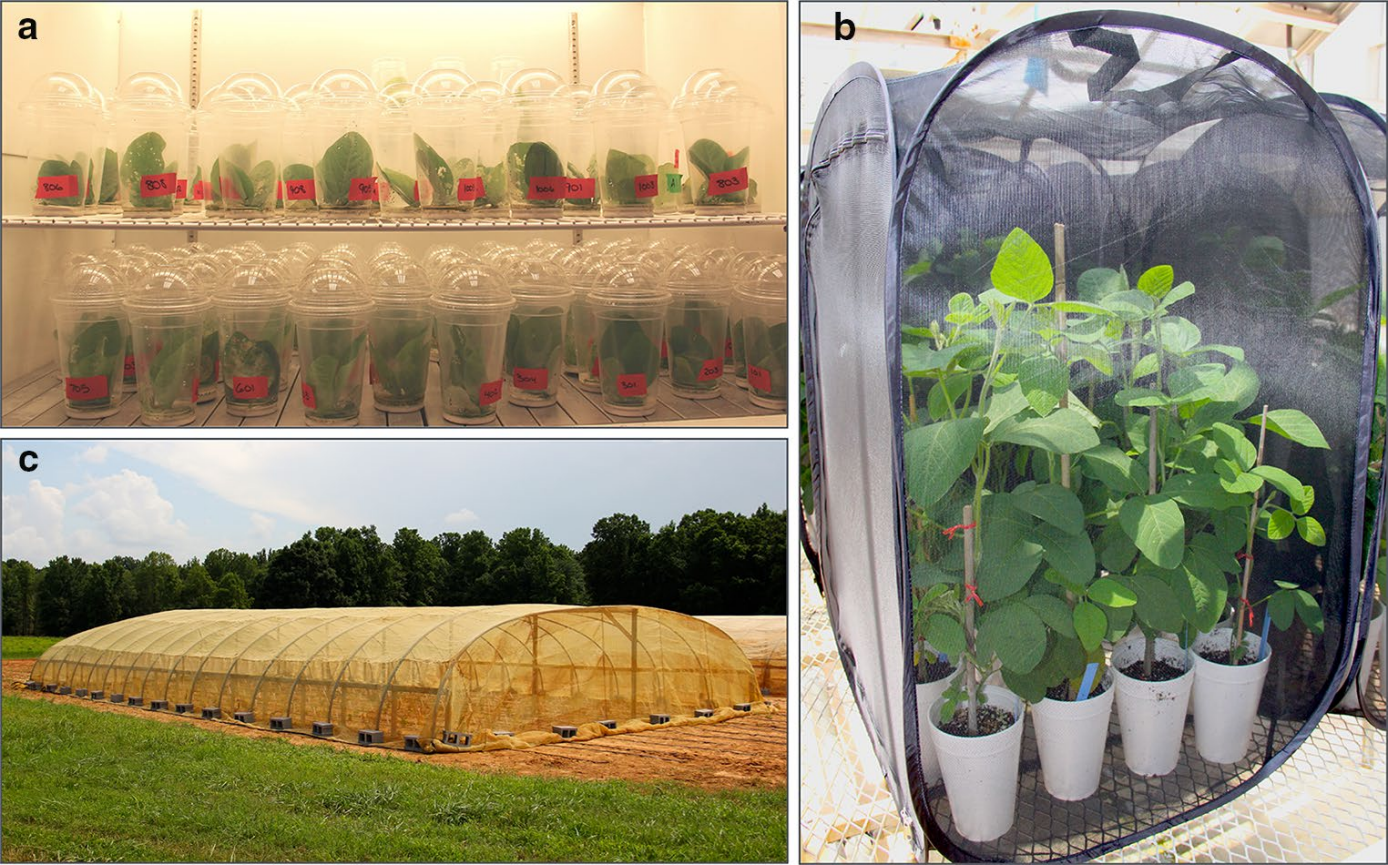
Insect bioassay settings: **a** Detached-leaf assay: caterpillars feeding on soybean leaves were contained in plastic cups. **b** Greenhouse assay: each cage contained caterpillars feeding on a block of test soybeans. **c** Cage built at the UGA Athens Plant Sciences farm to perform the field-cage assays

Antibiosis (non-choice) assays were used to determine the effect of the ME and MGHE QTL combinations on caterpillar weight gain. Benning (susceptible check), Benning^M^, Benning^E^, Benning^H^, Benning^G^, and Benning^MGH^ were included in each experiment. The NILs were tested for antibiosis to SBL, CEW, FAW, and VBC. Each species was evaluated independently using a randomized complete block design with 15 replications. Each replication included one plant from each genotype as the experimental unit. The experimental procedures included: (1) One seed was planted in a 450-ml polystyrene foam cup filled with Fafard 2 mix (Conrad Fafard, Agawam, MA, USA) with three holes punched in the bottom to provide drainage. Plants were grown in an insecticide-free greenhouse under a photoperiod of 16 h. Sunlight was supplemented with 400 J s^−1^ Phillips ED-18 high-pressure sodium lamps (Phillips Inc., Andova, MA, USA) to keep the plants in a vegetative stage. The temperature was regulated to approximately 28 °C during the day, and 20 °C at night. Newly expanded trifoliolate leaves were collected, once plants reached the V4 stage (Fehr and Caviness 1977). One trifoliolate leaf was placed into a 600-ml (20-oz) clear polystyrene cup (Letica Corporation) sealed with a dome lid (Letica Corporation). Each cup contained 7 ml of plaster of Paris saturated with water, to maintain 75 % relative humidity. Five SBL or FAW neonate caterpillars were placed in each cup, whereas only one CEW and VBC neonate was used per cup, with two cups per plant, to avoid cannibalism. Infested cups were placed in a growth chamber set at 27 °C, and a 14-h light period was maintained with fluorescent lights (T8 F032/730/Eco, Sylvania Octron, Danvers, MA, USA) providing ca. 40 µmol photons m^−2^ s^−1^ (Zhu et al. 2008) (Fig. 3a). Trifoliolate leaves were replaced with fresh leaves on the 4th day, and subsequently whenever 60 % of the leaf area had been consumed. The average percentage of defoliation was estimated based on the appearance of the entire leaf. The experiment was terminated after 7 days; caterpillars were immobilized by placing the cups at 4 °C for 24 h. Caterpillars from each cup were weighed and their mean weights were used for analysis of variance.

##### Greenhouse experiments

Antixenosis (choice) assays were used to evaluate caterpillars’ feeding preference when foliage of the null, M, E, H, G, ME, MGH, and MGHE NILs formed a canopy. The percentage of leaf area consumed by SBL, CEW, FAW, and VBC was determined for the each entire plant. Each insect species was tested independently using a randomized complete block design with 15 replications, with one plant from each NIL as the experimental unit. One seed was planted in a 450-ml polystyrene foam cup as described previously and grown in an insecticide-free greenhouse with the conditions as described above. Once plants reached the V4 stage, each block was transferred to a 24 × 24 × 36″ polyester-mesh cage (BioQuip products, Rancho Dominguez, CA, USA) (Fig. 3b). Each plant was infested with 10 neonate caterpillars. Since leaves of neighboring plants were in contact with each other, the caterpillars were able to move from plant to plant at will. Feeding was terminated when defoliation of Benning was higher than 50 %, which took approximately 10 days. Percent defoliation of each entire plant was estimated by at least three researchers, and the mean of the estimates for each plant was used for an analysis of variance.

##### Field-cage experiments

This assay was designed to evaluate resistance to SBL under field conditions; resistance was scored as percent defoliation, which includes the effects of antibiosis and antixenosis. A field-cage containing the null, M, E, H, G, ME, MGH, and MGHE NILs was installed at the University of Georgia Plant Sciences Farm (Fig. 3c). The experiment was planted on 1 July 2013 in a randomized complete block design with 15 replications. The experimental unit was a 6-plant hill plot (Bonnett and Bever 1947); each block contained one plot per NIL. Hills were spaced 76.2 cm apart and were thinned to six plants after germination. A single border row of Benning hill plots surrounded the experiment. After the plants reached the V2 stage, a cage covered with 0.9 × 0.9 mm Saran screen (Asahi Kasei, Tokyo, Japan) was placed over the experimental area. This confined the test insects and prevented immigration of parasitoids, predators, and other insect pests. The hill plots were infested when plants reached the V3 stage. Each hill plot was initially infested with 200 caterpillars. After that, 50 neonate caterpillars were added to the each hill plot twice a week for 2 consecutive weeks. The percent defoliation for each hill of plants was estimated by four researchers at 5, 7, 11, and 14 days after the first infestation. A second field cage containing the null, M, E, ME, MGH, and MGHE NILs was planted in 26 August 2013. This cage was infested and evaluated for defoliation as described for the first cage.

### Characterization of Benning^ME+*cry1Ac*^

#### Line development

**Fig. 4:**
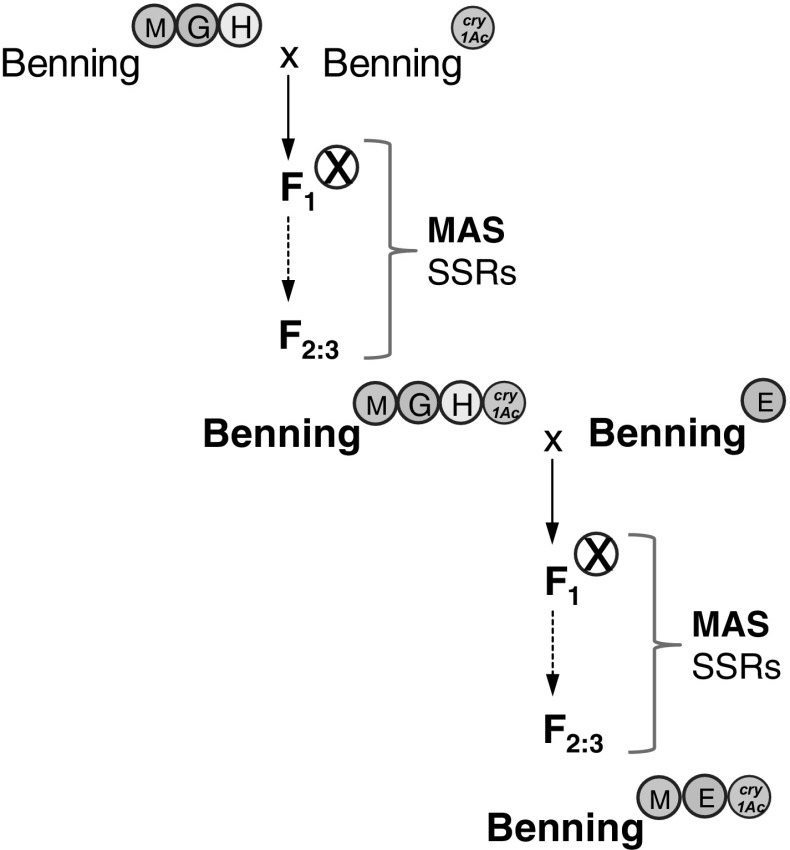
Breeding scheme for pyramiding insect-resistance QTLs and *cry1Ac* in Benning. SSRs were used for marker-assisted selection (MAS) of QTLs in each generation. SNPs (Ortega 2016, personal communications) were used to genotype the plants used in the phenotyping assays

The Benning^ME+*cry1Ac*^ line was developed from a cross between Benning^MGH^ and Benning^*cry1Ac*^ (Zhu et al. 2008); the breeding scheme is shown in Fig. 4. The presence of QTL-M and QTL-E was confirmed by genotyping for Sat_258 and Satt702, and for Sat_112 and Satt411, respectively. The presence of *cry1Ac* was confirmed by PCR, using the primers described by Stewart et al. (1996).

#### Cry1Ac toxin in leaf tissue

The *cry1Ac* and ME+*cry1Ac* plants were tested for *cry1Ac* expression using the Cry1Ab/Cry1Ac ImmunoStrip test (Agdia Inc., Elkhart, IN, USA). Two leaf punches were collected per plant. Samples were ground in 300 µl of SEB4 extraction buffer (Agdia Inc.) using a GenoGrinder 210 (Spex SamplePrep, Metuchen, NJ, USA). Leaf extracts were processed according to the manufacturer’s instructions.

#### Detached-leaf experiments

SBL, VBC, and southern armyworm (SAW) [*Spodoptera eridania* (Cramer)] were used in non-choice assays to determine the effect of the ME+*cry1Ac* pyramid on caterpillar weight gain. These species were chosen because they vary in their sensitivity to Cry1Ac; SBL and VBC are susceptible, while SAW is resistant (Bernardi et al. 2014b). Eggs were obtained from Benzon Research Inc. (Carlisle, PA, USA). In each assay, Benning, Benning^ME^, and Benning^*cry1Ac*^ were included as controls. The assays were set up and evaluated, as described in the previous section. Each assay consisted of a randomized complete block design with six replications. For the SAW assay, one cup containing five caterpillars was used to test each plant.

### Data analyses

Data recorded from antibiosis, antixenosis, and field-cage assays were analyzed using JMP statistical software version 10.0 (SAS Institute, Inc., Cary, NC, USA). Each dataset was tested for normality using the Shapiro–Wilk test (*P* > 0.05) (Shaphiro and Wilk 1965). A one-way ANOVA test (*P* > 0.01) was used to detect any difference among genotypes and experimental blocks, and a post hoc Tukey–Kramer multiple comparison test (*P* > 0.01) (Kramer 1956, 1957; Tukey 1953) was used to determine significant differences between genotypes.

## Results

### Characterization of Benning^ME^ and Benning^MGHE^

#### Detached-leaf experiments

**Fig. 5:**
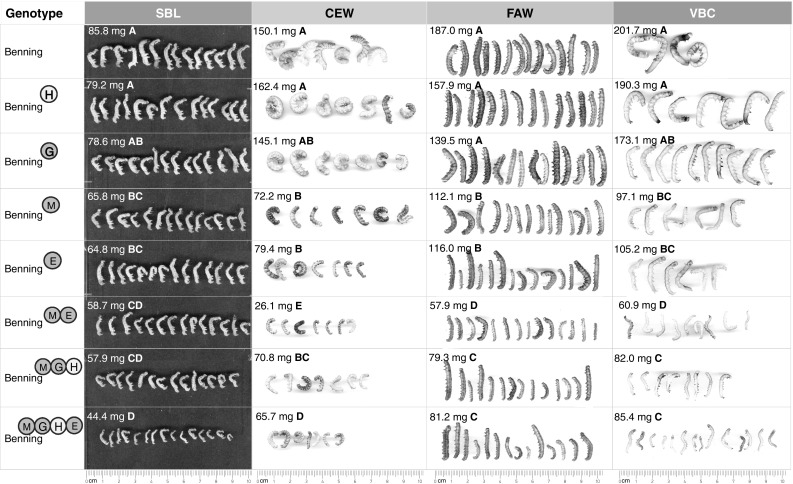
Mean weight of SBL, CEW, FAW, and VBC caterpillars after feeding on insect-resistant NILs during detached-leaf (antibiosis) assays. Significant differences (Tukey–Kramer post hoc test, *p* < 0.05) between NILs are indicated by *letters*

The results for the non-choice assays are shown in Fig. 5. MGHE had the strongest antibiotic effect against SBL; SBL feeding on Benning^MGHE^ was 48 % smaller than that feeding on Benning. However, ME had the strongest antibiotic effect against CEW, FAW, and VBC. CEW feeding on Benning^ME^ weighed 83 % less than CEW feeding on Benning. FAW feeding on Benning^ME^ weighed 69 % less than that feeding on Benning. Finally, VBC feeding on Benning^ME^ weighed 70 % less than VBC feeding on Benning. Lines carrying QTL-H and QTL-G did not show antibiosis to any of the insect species.

#### Greenhouse experiments

**Fig. 6:**
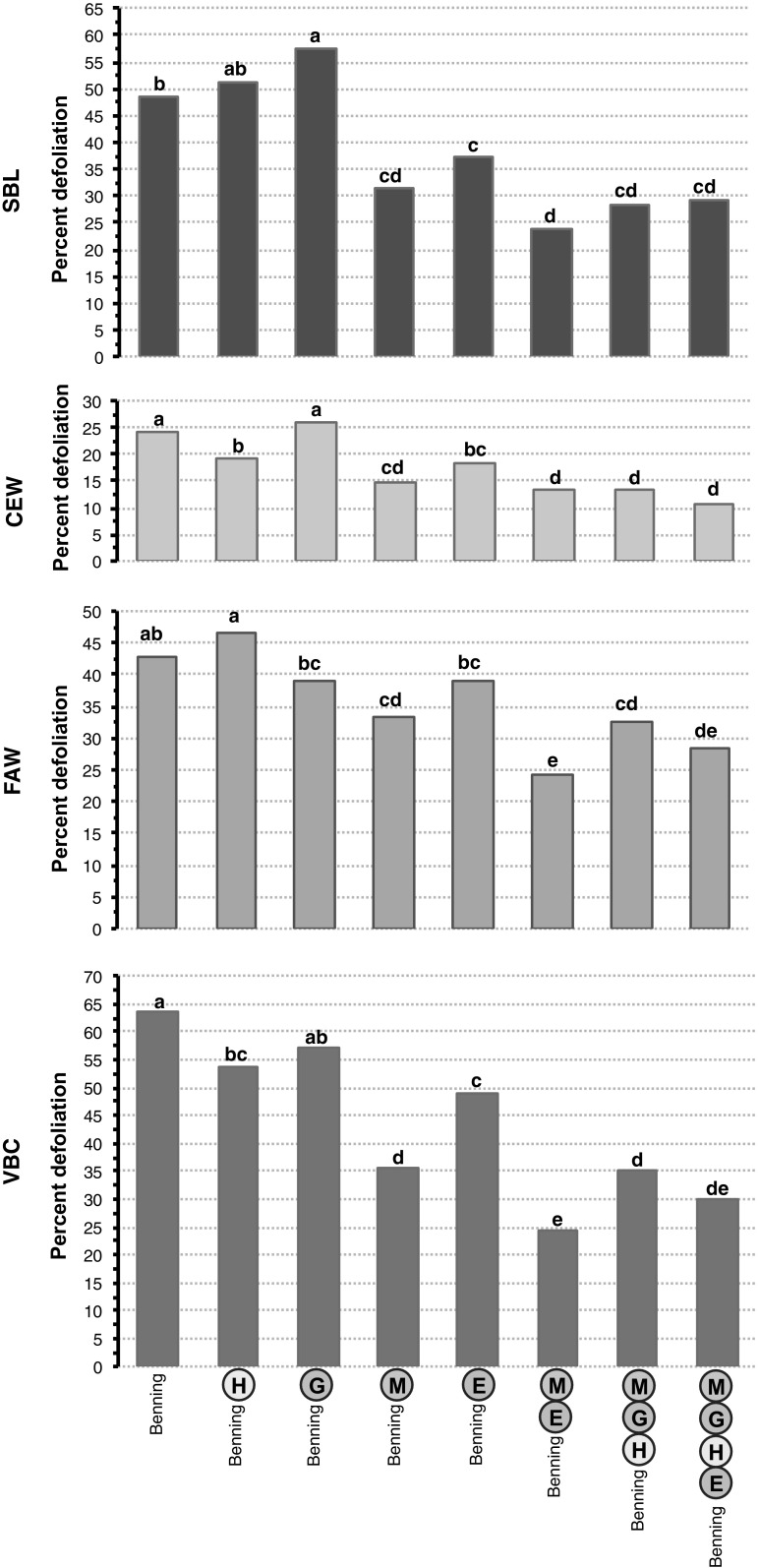
Mean defoliation by SBL, CEW, FAW, and VBC caterpillars on NILs during greenhouse (antixenosis) assays. Significant differences (Tukey–Kramer post hoc test, *p* < 0.05) between NILs are indicated by *letters*

Results for the SBL, CEW, FAW, and VBC choice assays are shown in Fig. 6. The pyramided NILs Benning^ME^, Benning^MGH^, and Benning^MGHE^ were the least defoliated across the four experiments. In the SBL and CEW bioassays, the combinations ME and MGHE were as resistant as MGH (*P* > 0.01). Benning^ME^ tended to have less SBL defoliation than Benning^MGH^ and Benning^MGHE^; however, this difference was not significant. Similarly, Benning^MGHE^ tended to have less CEW defoliation than Benning^ME^ and Benning^MGH^ (13.3 %). In the FAW and VBC bioassays, Benning^ME^ was more resistant than Benning^MGH^, but not significantly different from Benning^MGHE^ (*P* > 0.01).

Benning and Benning^G^ were the most susceptible lines averaged across experiments. Benning^H^ showed resistance to CEW and VBC assays; however, QTL-H alone failed to protect the plants from SBL and FAW caterpillars. Benning^M^ and Benning^E^ were the most resistant single-QTL NILs. QTL-M and QTL-E provided similar levels of resistance against SBL, VBC, and FAW. Nonetheless, Benning^M^ was significantly more resistant against VBC than Benning^E^.

#### Field-cage experiments

**Fig. 7:**
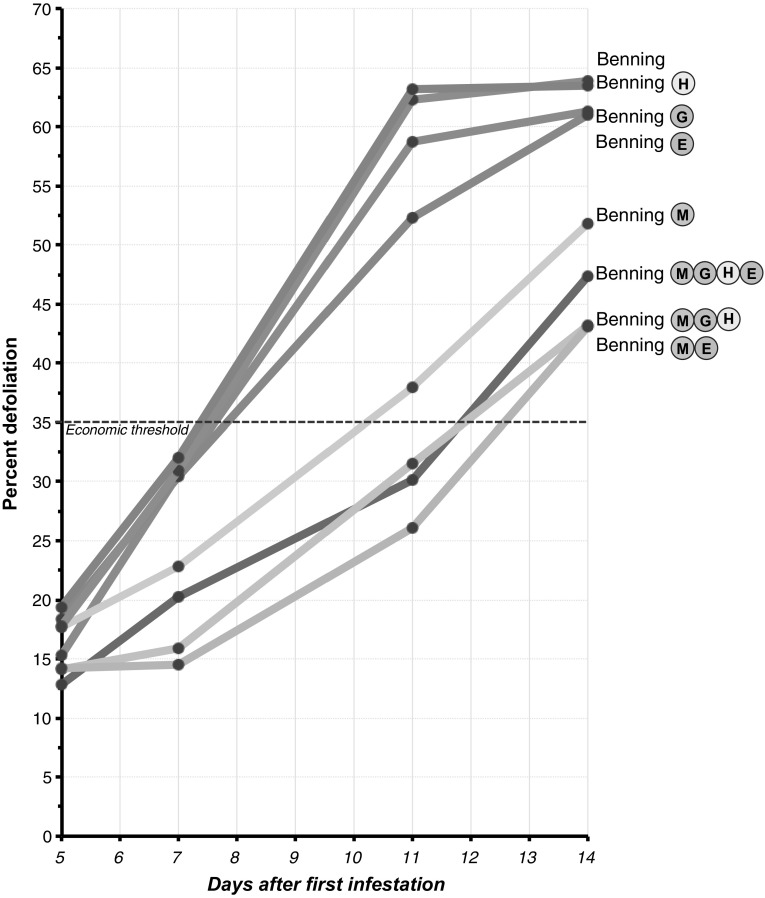
Feeding progression of SBL in the first field cage. Percentage of defoliation per hill was recorded at 5, 9, 11, and 14 days after the first infestation. Each time point shows the mean defoliation per NIL

**Fig. 8:**
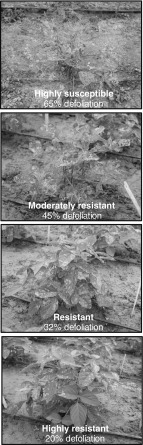
Leaf damage on NILs exposed to SBL feeding in the field cage, at 11 days after infestation

*Defoliation progression in cage 1.* The mean percentage of defoliation on each NIL at 5, 7, 11, and 14 days after infestation is shown in Fig. 7. At 5 days, defoliation ranged between 12 and 18 %, and no significant differences were observed between the NILs. At 7 days, Benning showed the most defoliation (32 %) and Benning^ME^ was the least defoliated (14 %). At this time point, caterpillars were actively moving between hills, and towards the Benning hills used as borders. At 11 days, susceptible and resistant hills were easily distinguishable (Fig. 8); Benning still showed the most defoliation (63 %) and Benning^ME^ was the least defoliated (26 %). At day 14, the rate of feeding was significantly slower; few caterpillars had migrated to the resistant NILs, but the majority of them were located on the cage’s mesh.

**Fig. 9:**
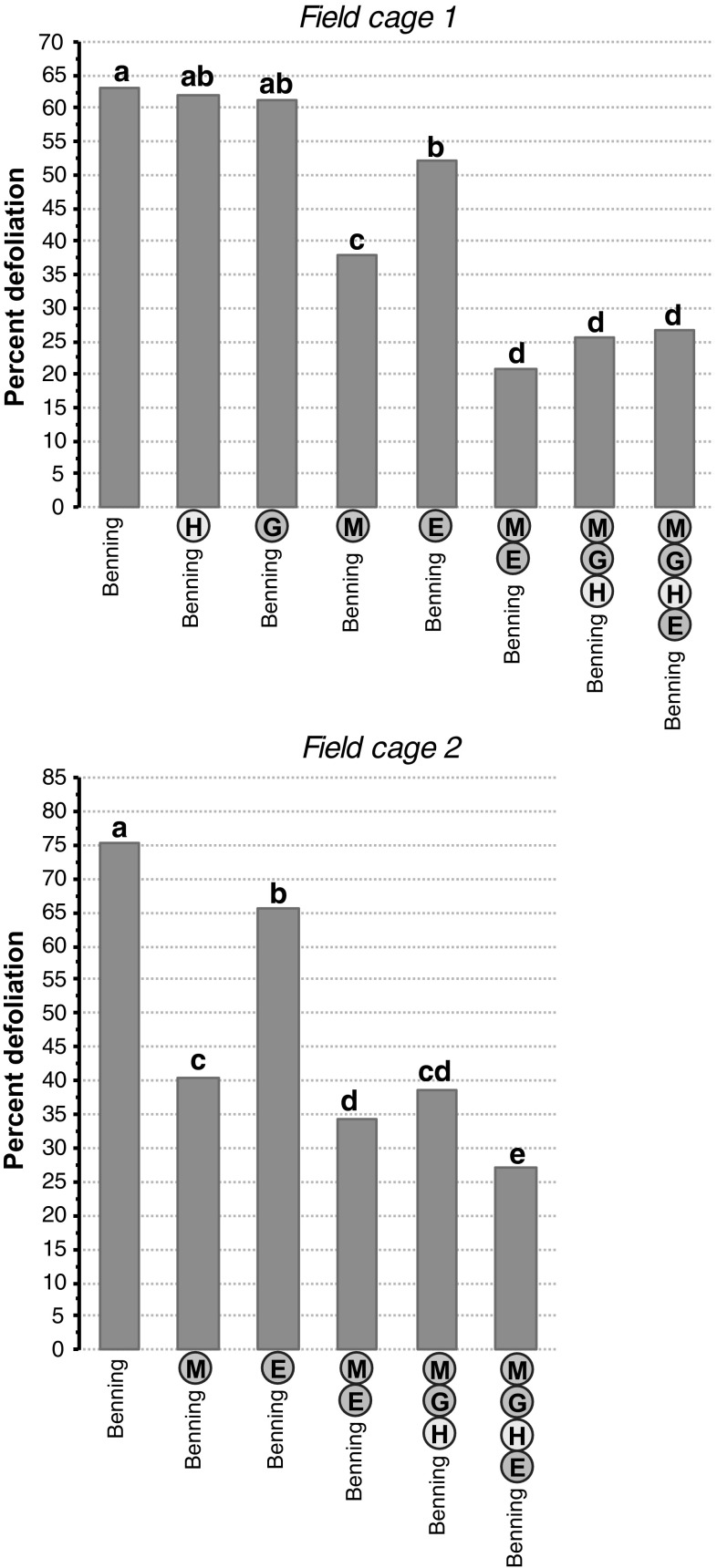
Mean defoliation by SBL at 11 days after infestation, in the first and second field cage. Significant differences (Tukey–Kramer post hoc test, *p* < 0.05) between NILs are indicated by *letters*

*Defoliation in cage 1.* The data collected at 11 days after infestation were analyzed to determine differences in levels of resistance among NILs. This time point was selected, because the plants were highly defoliated and the caterpillars were still highly active. Benning^ME^ (21 %), Benning^MGH^ (25 %), and Benning^MGHE^ (27 %) were the most resistant lines in this cage, followed by Benning^E^ (52 %) and Benning^M^ (38 %), which were moderately resistant. Benning (63 %) Benning^H^ (62 %), and Benning^G^ (61 %) were the most susceptible (Fig. 9a).

*Defoliation in cage 2.* Benning^G^ and Benning^H^ were excluded, because in the first cage they were not resistant to SBL. Benning^MGHE^ (27 %) was the most resistant line in this cage, followed by Benning^ME^ (34 %) and Benning^MGH^ (39 %). Benning^E^ (65 %) and Benning^M^ (45 %) were more defoliated than Benning^ME^ and Benning^MGH^ in this cage; however, Benning^E^ and Benning^M^ were less defoliated than Benning (75 %) (Fig. 9b).

### Characterization of Benning^ME+c*ry1Ac*^

#### Detached-leaf experiments

**Fig. 10:**
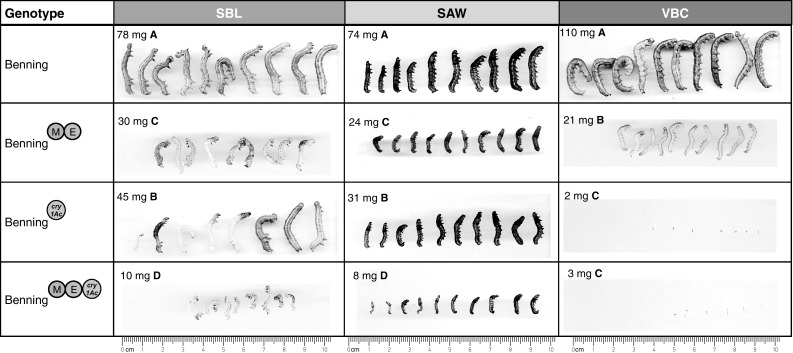
Mean weight of SBL, SAW, and VBC caterpillars after feeding on insect-resistant Benning^ME+*Cry1Ac*^ during detached-leaf (antibiosis) assays. Significant differences (Tukey–Kramer post hoc test, *p* < 0.05) between NILs are indicated by *letters*

The results for the non-choice assays are shown in Fig. 10. The pyramid of QTL-M, QTL-E, and *cry1Ac* showed enhanced antibiosis against SBL and SAW when compared to Benning^ME^ and Benning^*cry1Ac*^. SBL fed on Benning^ME^ and Benning^*cry1Ac*^ weighed 61 % and 43 % less than SBL fed on Benning. However, the strongest antibiotic effect against SBL was observed in Benning^ME+*cry1Ac*^; these caterpillars weighed 88 % less than Benning-fed caterpillars. SAW fed on Benning^ME^ and Benning^*cry1Ac*^ weighed 68 % and 59 % less than SAW fed on Benning. The strongest antibiotic effect against SAW was observed on Benning^ME+*cry1Ac*^; these caterpillars weighed 89 % less than those fed on Benning. VBC fed on Benning^ME^ weighed 81 % less than VBC fed on Benning. VBC fed on Benning^*cry1Ac*^ died at the first instar; their weight was 98 % less than Benning-fed VBC. VBC fed on Benning^ME+*cry1Ac*^ also died at the first instar; therefore, the effect of QTL-M and QTL-E could not be measured for this species.

## Discussion

PI 229358 and PI 227687 have been used in soybean breeding programs worldwide to introgress resistance to chewing insects. This is the first time that the resistance of NILs carrying pyramids of insect-resistance QTLs from PI 229358 and PI 227687 has been evaluated. The rationale was based on work by Lambert and Kilen (1984a), showing that F_1_ progeny from PI 229358 x PI 227687 are more resistant than either parent. In this study, it was demonstrated that the QTL combinations ME and MGHE are able to confer high levels of resistance against multiple insect species via antibiosis and antixenosis, in the cultivar, Benning. The ME and MGHE NILs exhibit similar levels of resistance in all but one of the bioassays. Therefore, there is no indication that the addition of QTL-G and/or QTL-H to the ME combination is required to reach agriculturally relevant levels of resistance. Although the results of are encouraging, a limitation of this study might be that ME and MGHE were characterized in a single genetic background (Benning), due to the time and resources needed to develop the NILs. Nevertheless, QTLs M (Narvel et al. 2001; Walker et al. 2002; Walker et al. 2004) and E (Hulburt 2002; Hulburt et al. 2004) have been verified to work in different backgrounds when independently tested. From a breeding perspective, introgressing just QTL-M and QTL-E into an elite cultivar is simpler than introgressing all four QTLs. As the number of QTLs increases, pyramiding in an elite line becomes increasingly difficult; especially when selection involves several traits at a time (Bernardo 2008). Furthermore, QTL-G is associated with a yield penalty (Warrington 2006). Altogether, pyramiding the major insect-resistance QTLs from PI 229358 and PI 227687 presents an effective genetic combination to deploy host–plant resistance to insects in soybean.

In Brazil, the genetically modified MON 87701 × MON 89788 soybean, which expresses the Bt toxin Cry1Ac, is used for the integrated pest management of lepidopteran pests (Berman et al. 2011). This soybean is resistant to SBL, VBC (Bernardi et al. 2012), tobacco budworm [*Heliothis virescens* (Fabricius)] (Bernardi et al. 2014a), and the recently imported old world cotton bollworm [*Helicoverpa armigera* (Hübner)] (Azambuja et al. 2015). However, Cry1Ac is not sufficient to protect soybeans from FAW, SAW, and the velvet armyworm [*Spodoptera latifascia* (Walker)] (Bernardi et al. 2014b). Frequent SAW outbreaks have been already reported in Brazil (Bueno et al. 2007; Santos 2005); SAW’s high defoliation capacity (Bueno et al. 2011) and its large populations make this species an important pest that can cause severe economic losses to Brazilian soybean production. A synergistic relationship between *cry1Ac* and the insect-resistance QTLs from PI 229358 was previously reported (Walker et al. 2002; Zhu et al. 2008). PI 227687 has shown resistance to SAW via antibiosis (Souza et al. 2014). There was interest in determining if the combination of QTL-M, QTL-E and *cry1Ac* would also provide enhanced resistance to lines with only the *cry1Ac* transgene or the QTLs by themselves. Benning^ME+*cry1Ac*^ was developed and characterized in antibiosis assays. This line is more resistant than Benning^ME^ and Benning^*cry1Ac*^ against SBL and SAW. Although this combination would need to be thoroughly studied in antixenosis field-cage assays and, if possible, in field tests with natural pest infestations, the results from the antibiosis assays indicate the potential of combining QTL-M, QTL-E and *cry1Ac* to improve soybean resistance to insects that are naturally tolerant to *cry1Ac*. The use of this pyramid as part of a resistance management strategy (Bates et al. 2005) could help preserve the effectiveness of Bt, which could lead to durable resistance to leaf-chewing insects in soybean.

Breeding high-yielding soybean cultivars with agriculturally relevant levels of insect-resistance has been a long-term goal. In the past, lines carrying only PI 229358 QTLs were either lower yielding (e.g., Benning^MGH^; Warrington et al. 2008), or not highly resistant in the field (e.g., Benning^MH^; Zhu et al. 2008). With only two insect-resistance QTLs, Benning^ME^ is at least as resistant to several important lepidopteran pests as Benning^MGH^, without carrying QTL-G. Lines carrying QTLs from PI 229358 QTLs enhance the resistance provided by *cry1Ac* in lines like Benning^MH+*cry1Ac*^ (Zhu et al. 2008). The combination of ME+*cry1Ac* described here could present an opportunity to effectively deploy Bt, in a pyramid with host–plant resistance genes.

### Author contribution statement

MO designed and performed the experiments, collected and analyzed data, and drafted the manuscript. JA and RB conceived the study. WP conceived the study and drafted the manuscript.

## Abbreviations

IPM: Integrated pest management
SSR: Simple sequence repeat
SNP: Single nucleotide polymorphism
Chr: Chromosome
cM: Centimorgans
bp: Basepair
PI: Plant introduction
QTL: Quantitative trait locus
CEW: Corn earworm
SBL: Soybean looper
VBC: Velvetbean caterpillar
FAW: Fall armyworm
SAW: Southern armyworm
Bt: *Bacillus thuringiensis*
